# Inhibit My Disinhibition: The Role of the Inferior Frontal Cortex in Sexual Inhibition and the Modulatory Influence of Sexual Excitation Proneness

**DOI:** 10.3389/fnhum.2018.00300

**Published:** 2018-07-26

**Authors:** Geraldine Rodriguez, Alexander T. Sack, Marieke Dewitte, Teresa Schuhmann

**Affiliations:** ^1^Brain Stimulation and Cognition Laboratory, Department of Cognitive Neuroscience, Maastricht University, Maastricht, Netherlands; ^2^Department of Clinical Psychological Science, Maastricht University, Maastricht, Netherlands

**Keywords:** sexual inhibition, sexual excitation, inferior frontal gyrus, brain stimulation, approach-avoidance, negative priming, response inhibition

## Abstract

Sexual behaviour is the result of an interplay between distinct neural inhibitory and excitatory mechanisms. Individual differences in sexual excitation and sexual inhibition are proposed to play an important role in the processes sustaining the regulation of sexual behaviour. While much research has focused on the neural correlates of response inhibition, highlighting a prominent role of the inferior frontal gyrus (IFG), very little is known regarding the neural mechanisms underlying different aspects of sexual inhibition. Here, we experimentally combined functional magnetic resonance imaging (fMRI) and transcranial magnetic stimulation (TMS) to: (i) test the functional role of IFG during motivational and cognitive sexual inhibition; and (ii) reveal whether this IFG involvement in sexual inhibitory processes depends on sexual excitation and sexual inhibition as traits. Twenty-two participants performed an Approach-Avoidance (AA) and a Negative Affective Priming (NAP) paradigm to assess motivational and cognitive sexual inhibition respectively. Our fMRI study showed IFG being selectively activated during cognitive but not motivational sexual inhibition. Importantly, the level of this neural activity was modulated by individual sexual excitation scores. Interestingly, a transient disruption of IFG activity using TMS led to an improvement in cognitive, not motivational, sexual inhibition, but only when accounting for individual sexual excitation scores. These findings clearly document that sexual excitation modulates IFG activity levels during cognitive sexual inhibition, and at the same time determines the effects of TMS on IFG by improving cognitive control exclusively for individuals with high sexual excitation scores. These findings provide new insights regarding the functional role of IFG, and underscore the relevance of individual psychological differences in understanding the brain mechanisms underlying socioaffective processes.

## Introduction

Sexuality is evolutionary relevant and individually rewarding and thus constitutes one of the leading motivating forces in human behaviour. Since sexual arousal can arise fairly automatically in response to sexual stimuli that are omnipresent in everyday life, adequate social behaviour requires the ability to inhibit sexual responses that potentially harm social norms and individual well-being.

The ability to inhibit unfolding sexual responses comprises different psychological and physiological mechanisms that occur in parallel with those processes that elicit sexual arousal. According to the dual control model of sexual response, any form of sexual behaviour is the consequence of the interplay and balance between mechanisms that activate and facilitate the unfolding of the sexual response (sexual excitation) and mechanisms that diminish or avoid this response (sexual inhibition). Sexual inhibition and sexual excitation cannot only be studied as a process but also as traits, as individuals differ in their propensity to become sexually aroused or inhibited (Bancroft and Janssen, 2000). According to the dual control model of sexual response, individuals with high sexual excitation and low sexual inhibition have an increased propensity to engage in inadequate sexual behaviour.

Individual variabilities in sexual excitation and sexual inhibition have been largely studied with the Sexual Excitation/Sexual Inhibition Scales (SES/SIS; Bancroft et al., 2009). Higher levels of sexual excitation have repeatedly been observed in individuals with out-of-control sexual behaviour (Bancroft and Vukadinovic, 2004; Janssen and Bancroft, 2007; Winters et al., 2010; Rettenberger et al., 2016). On the other hand, sexual inhibition has shown to be weakly or not related to hypersexual behaviours (Miner et al., 2016; Rettenberger et al., 2016), but has shown to be lower in individuals who engage more in risky sexual practices (Bancroft et al., 2009).

As sexual arousal can impact cognition and the control of sexual behaviour (Ariely and Loewenstein, 2006), it is expected that individuals who are more easily sexually aroused also differ in their sexual inhibition processing. Macapagal et al. (2011), for example, showed that individuals high in sexual excitation (as measured by the SES) committed more omission mistakes during a sexual Go/No-go paradigm, which is a measure of sexual response inhibition, after watching an erotic film than individuals low in sexual inhibition. Sexual inhibition (as measured by the SIS) did not relate to omission or comission mistakes, presumably because the SIS tackles the inhibition due to potential negative consequences of sexual activity, which was not included in the experiment design (Macapagal et al., 2011). Thus, although sexual inhibition traits may influence complex socio-sexual behaviour (e.g., risky sexual practices), individual differences in sexual excitation seem to have more predictive value for understanding basic sexual inhibitory processing and their neural mechanisms.

In contrast to the large body of evidence regarding the neural mechanisms of sexual arousal (see Stoléru et al., 2012 for a review), much less is known about the neural mechanisms underlying sexual inhibition. A pionering study by Beauregard et al. (2001), aimed to characterise the neural network engaged when individuals deliberately regulate their sexual arousal. Participants were explicitly asked to inhibit their sexual arousal while watching erotic stimulus material. In comparison to passively viewing the same stimuli, an enhanced anterior cingulate activity, left inferior frontal gyrus (IFG) and the right superior frontal gyrus activity was observed during the attempted sexual inhibition (Beauregard et al., 2001). Interestingly, the IFG has also been proposed to be a tonic inhibitor of sexual arousal as it was deactivated during the passive viewing of sexual stimuli (Redouté et al., 2005).

These studies provide converging evidence that the IFG, is associated with sexual inhibition. This region has also been associated with different types of more classic response inhibition paradigms (e.g., Go/No-go and Stop Signal task; Aron et al., 2014), self-regulation (Cohen and Lieberman, 2010; Tabibnia et al., 2011) and with exerting top-down control over rewarding stimuli (Goldstein and Volkow, 2011). Recent models have challenged this dominant view of the IFG being mainly an inhibitory module, arguing for its common role in multiple cognitive demands such as task switching or salience detection which are inherent to inhibitory paradigms (Erika-Florence et al., 2014; Hampshire and Sharp, 2015).

In the field of sexual cognition, the IFG is one of the regions that are more actively engaged during the passive viewing of erotic stimuli in hypersexual individuals as compared to controls (Voon et al., 2014; Seok and Sohn, 2015). The IFG has also been related to the amount of penile tumescence in response to sexual images (Moulier et al., 2006), and to levels of sexual addiction (Seok and Sohn, 2015). In addition, IFG activation in response to sexually appealing stimuli has shown to depend on testosterone levels in male participants (Stoléru et al., 1999; Redouté et al., 2005) and on the menstrual phase in females (Roberts et al., 2008; Zhu et al., 2010). These studies suggest that in addition to its potential role for sexual inhibition and control, also sexual excitatory mechanisms modulate activity in IFG during the perception of sexual stimuli. This apparent contradiction, i.e., IFG being activated during sexual inhibition while at the same time responding stronger to sexually arousing stimuli, may become plausible when considering that the higher the arousal experienced by a given individual, the higher the demand for inhibitory control. This would also explain why this increased IFG response to arousing stimuli is dependent on, e.g., testosterone levels or levels of sexual addiction as a trait.

In the current study, we aim to directly investigate whether the IFG is causally engaged in sexual inhibition, and whether this potential functional engagement of the IFG is dependent on sexual excitation and/or sexual inhibition proneness, under the assumption that individuals that are more prone to be sexually aroused or less prone to inhibit their sexual response, have different cognitive demands when trying to inhibit their sexual thoughts and behaviour. Two sexual paradigms were used to assess sexual inhibition: the Negative Affective Priming (NAP) paradigm assessing the cognitive component of inhibitory control, and the Approach-Avoidance (AA) paradigm designed to involve a motivational-motor driven component of sexual inhibition. Previous research has shown that both paradigms were sensitive to predict the frequency of sexual thoughts and pornography watching respectively, and represent two independent inhibitory mechanisms (Rodriguez et al., in press). Functional magnetic resonance imaging (fMRI) was combined with transcranial magnetic brain stimulation (TMS) in two separate experimental studies. In Experiment 1, participants performed the NAP and AA while their task-related brain activity changes were measured using fMRI, identifying the exact brain regions specifically involved in sexual inhibition. We expected sexual inhibitory processes to be intrinsically connected to the extent to which individuals are prone to being sexually aroused and/or inhibited, accordingly, we hypothesised that individual differences in sexual excitation and/or sexual inhibition (measured by the SES/SIS; Janssen et al., 2002) would modulate the IFG neural response during sexual inhibition. In the second experiment, we tested whether the revealed brain activations are essential in encoding for the inhibitory process itself by assessing the behavioural effects of disrupting IFG activity with TMS in the NAP and AA paradigms. We hypothesised that the IFG dysruption effect might be modulated by individual differences in sexual excitation and/or sexual inhibition. Finally, in both studies, we included a classic Go/No-go paradigm as a non-sexual inhibitory reference task, which has consistently shown to also recruit the IFG in the context of general response inhibition (Levy and Wagner, 2011; Aron et al., 2014; Dambacher et al., 2014).

## Experiment 1: Functional Magnetic Resonance Imaging (fMRI) to Identify Brain Regions Activated During Sexual Inhibition

### Method

#### Participants

Twenty-four healthy male participants (18–34 years old) with no neurological or psychiatric disorders took part in this study. One participant was excluded due to extensive head movements and a second participant due to technical difficulties leading to an incomplete data set (final sample: *N* = 22, mean age = 24.77, SD = 4.76). All participants received a written description of the experiment and the relevant practicalities about the use of MRI prior to accepting to participate. They gave written informed consent and were financially reimbursed for their participation. The study was approved by the local Ethical Committee of the Faculty of Psychology and Neuroscience at Maastricht University.

#### Design

The experiment consisted of one session in which participants performed two sexual (AA and NAP) and one non-sexual (Go/No-go) inhibition paradigm inside the MRI scanner. The order in which the two sexual tasks were presented was counterbalanced. The Go/No-go task was taking place between the sexual tasks to prevent habituation to sexual stimuli. At the end of the session participants filled out the computerised self-reports (SES/SIS; see description below).

#### Paradigms

##### Approach-Avoidance Task

**Figure 1:**
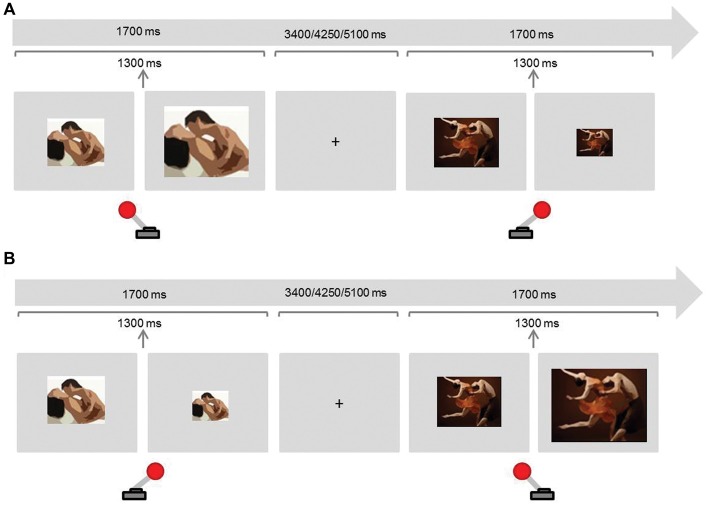
Experimental paradigm Approach-Avoidance (AA) task (motivational sexual inhibition). Participants were instructed to approach (pull the joystick towards them) sexual stimuli and avoid (push the joystick away) non-sexual stimuli in half of the blocks **(A)**, and to avoid sexual stimuli and approach sexual stimuli in the remaining blocks **(B)**. Approaching and avoiding stimuli produced a respective doubling or halving of the images.

To address *motivational sexual inhibition*, we adapted the AA task using sexual and neutral stimuli (adapted from Dewitte, 2016; Figure 1). Similar versions have shown to be related to the amount of viewing time of erotic stimuli (Hofmann et al., 2009), to be sensitive to gender differences (Dewitte, 2016) and to predict pornography watching frequency (Rodriguez et al., in press).

There were two congruent and two incongruent blocks. In congruent blocks participants were asked to approach sexual stimuli and avoid non-sexual stimuli whereas they were asked to do the opposite in the incongruent blocks. To approach a stimulus, participants pulled a joystick towards them which doubled the image size. To avoid, participants pushed the joystick away from them, which in turn halved the image size. The block order was counterbalanced across participants. Each block contained 24 sexual and 24 non-sexual randomised stimuli. Sexual stimuli were colour photographs displaying dyadic heterosexual intercourse or oral sex. In order to avoid habituation and the impact of individual sexual preferences, we aimed to present each stimulus only once for every condition. Stimuli were drawn from a previously evaluated and validated dataset (Rupp and Wallen, 2007). As not enough photographs were available, 5% were selected from the internet and evaluated by three judges to assure that the content and arousing properties were comparable to the previously validated pictures. Non-sexual stimuli were colour photographs of one woman and one man dancing (Dewitte, 2016). The proportion of the bodies’ dimensions (with particular attention to the female body) with respect to the whole picture was comparable in both conditions. Images were displayed on a light grey background and the default size of the image was 3379 × 2725 pixels (horizontal orientation) in half of the blocks and 2576 × 400 pixels (vertical orientation) in the other half. The presentation of the stimulus in each trial lasted 1700 ms and these were intercalated with fixation crosses presented for 3400, 4250, or 5100 ms. The resizing of the image took place after 1300 ms of the image presentation to avoid variations according to the response reaction times.

##### Negative Affective Priming Task

**Figure 2:**
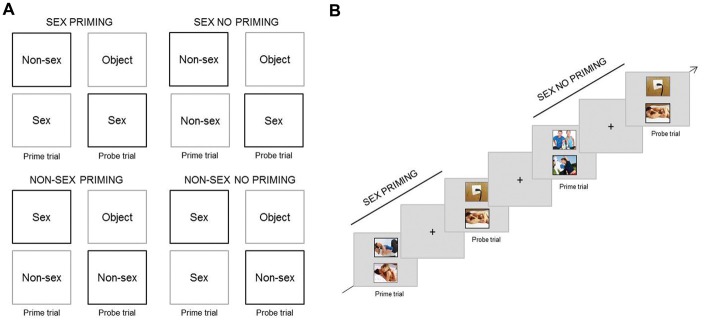
Experimental paradigm Negative Affective Priming (NAP) task (cognitive sexual inhibition). **(A)** Design of the four different trial-sequences (Stimuli [Sex vs. Non-sex] × Type [Priming vs. No Priming]). In priming probe trials participants had to respond towards the stimuli type (in black) that they ignored in the prime trial (in grey) of the same sequence. In no priming trials, the target content type (in black) in the probe trial did not match the distractor (in grey) in the prime trial. **(B)** Example of a Sex Priming sequence followed by a Sex No Priming sequence.

This task was selected to target *cognitive sexual inhibition* (adapted from Dewitte, 2016; Figure 2). The inhibition process is assessed through the negative priming effect, which consists in a delay of a response towards a stimulus that has been previously inhibited (see description below). This phenomenon is not noticeable to the participant and, therefore, is believed to measure automatic inhibition (for an overview on automaticity see Moors and De Houwer, 2006). The priming effect has shown to be larger for sexual than for neutral stimuli presumably due to a major implication of inhibition (Dewitte, 2016; Rodriguez et al., in press). This task predicted the frequency of sexual thoughts in daily life (Rodriguez et al., in press).

There were four types of trial-sequences: (a) Sex Priming; (b) Sex No Priming; (c) Non-Sex Priming; and (d) Non-Sex No Priming. A trial-sequence consisted of a prime trial and a probe trial; during each, two pictures were presented simultaneously. The pictures were displayed one above the other, and one was surrounded by a black frame and the other by a grey frame. The instruction was to attend only to the black frame picture (target), therefore ignoring the grey frame one (distractor) and to indicate whether the target displayed sexual or non-sexual content through button pressing. During the priming trial-sequences, the content type of the distractor in the prime trial matched the content of the target picture in the probe trial. In the control trial-sequences (No Priming) the content type of the distractor in the prime trial was different from the target in the probe trial. The target of the probe trial could be sexual or non-sexual. Figure 2A provides an overview of the four different conditions (Sex—Non-Sex × Priming—No Priming).

The four types of trial-sequences were presented randomly in equal proportions throughout each of three blocks. Each block contained 32 trial-sequences. The prime and probe trials were presented for 1700 ms each and were separated by a fixation cross displayed for the same duration. The probe and prime trials from different sequences were separated by the same fixation cross which lasted for 3400, 4250, or 5100 ms (Figure 2B). Sexual stimuli were pictures (320 × 260 pixels) different from those used in the AA task but with the same content characteristics. The non-sexual stimuli were colour photographs of one man and one woman exercising together. The neutral stimuli (distractors in the probe trials) were pictures of neutral objects (e.g., pencil case). Pictures were displayed on a light grey background and the picture frames were three pixels in width. Eighty-five percentage of photographs were selected from Rupp and Wallen (2007) and Dewitte (2016), and the remaining ones were selected from the internet, for the same reasons and on the same criteria basis as those selected for the AA task.

##### Go/No-Go Task

**Figure 3:**
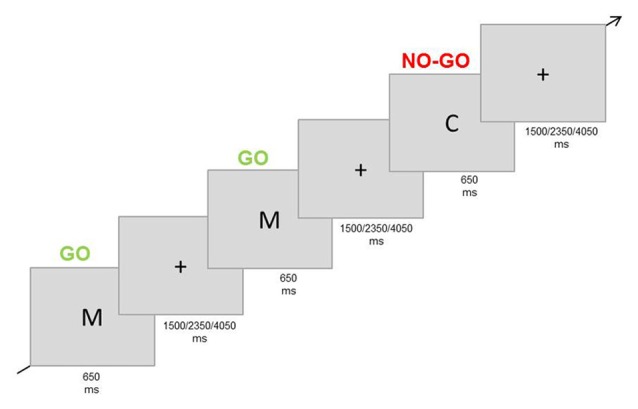
Experimental paradigm Go/No-go task (non-sexual inhibition task). Participants were asked to respond to a frequent stimulus (letter “M”) and to refrain from responding when the infrequent stimulus was presented (letter “C”).

This paradigm was used to target *general inhibition*. Participants were instructed to respond to a frequent Go stimulus and to not respond to an infrequent No-go stimulus. They responded with the right index finger on a button-box (Figure 3). As stimuli, the letters “C” and “M” were used and which letter was defined as the Go or No-go stimulus was counterbalanced across participants. Participants had to complete four blocks of 80 trials each (25% No-go trials). Every trial consisted of the presentation of the stimulus for 200 ms, followed by an inter-trial interval of 1500, 2350, or 4050 ms (Figure 3). Responses after 650 ms with respect to stimulus onset were not registered. The letters (3 × 2.3 cm) were displayed in white colour on a grey background (adapted from Dambacher et al., 2014).

Before entering the MRI room, participants performed 20 practice trials for the AA task, eight trial sequences for the NAP task, and 10 for the Go/No-go task. The number of practice trials was decided according to the complexity of every task. The practice trials involved different stimuli than the actual tasks to avoid habituation (animals and plants for the sexual tasks and “T” and “K” letters for the Go/No-go task). All tasks described in this manuscript were programmed and presented with PsychoPy software (Peirce, 2007).

#### Questionnaires

##### Sexual Inhibition/Sexual Excitation Scales (SIS/SES)

This 45 items-scale measures the individual propensity for sexual inhibition and excitation. It contains one factor quantifying sexual excitation and two factors quantifying sexual inhibition: (a) SIS1—inhibition derived from threat of sexual performance failure, distraction or lack of physical stimulation (14 items); and (b) SIS2—inhibition due to the threat of performance consequences, such as risk of being caught, unwanted pregnancy, sexually transmitted diseases, feeling or causing pain, and partner’s too young age (11 items). Answers were registered on a four-point Likert scale (Janssen et al., 2002). Previous studies showed solid internal consistency and test-retest reliability for the factors SES, SIS1 and SIS2 (Janssen et al., 2002; Current study: SES Cronbach’s alpha = 0.82; SIS1—Cronbach’s alpha = 0.73; SIS2—Cronbach’s alpha = 0.74).

#### Technical Details and fMRI Acquisition

Participants performed all paradigms inside the MRI Scanner as described above. Data were acquired at the 3T Siemens Prisma Scanner at the Maastricht Brain Imaging Center, Maastricht University. Functional EPI images were collected using an in-house developed multi-echo multi-band sequence (Poser et al., 2006; TR = 850 ms, TE = 15/30/44 ms, flip angle = 50°, FOV = 210 mm, 36 slices, isovoxel 3 mm^3^). Online-scanner reconstruction was performed using the slice-GRAPPA algorithm (Setsompop et al., 2012) with leakage artifact reduction (Cauley et al., 2014) as implemented in the reconstruction of the MGH blipped-CAIPI SMS-EPI distribution (software and complete documentation are available at https://www.nmr.mgh.harvard.edu/software/c2p/sms). The echo images were combined using an optimised echo weighting method as described in Poser et al. (2006). This GRAPPA sequence was used to optimise the BOLD signal in frontoventral regions.

High-resolution anatomical images were acquired with an MPRAGE sequence (TR = 2250 ms, TE = 2.21 ms, FOV = 256 mm, 192 sagittal slices, isovoxel 1 mm^3^).

#### fMRI Analyses

The imaging data were pre-processed and analysed with Brain Voyager QX Version 2.8.4.2645 (Brain Innovation, Maastricht, Netherlands). The images were motion-corrected (trilinear / sinc interpolation and aligned to the first functional volume acquired after the anatomical sequence) and corrected for slice timing skew using temporal sinc interpolation. A temporal high pass filter (three cycles) was applied. Images were co-registered to the individual T1 weighted images and normalised to Talairach stereotaxic space. Volume time courses were spatially smoothed using a 6 mm full width half maximum Gaussian kernel.

We conducted a random-effects general linear model (GLM) analysis for every task. An event-related approach was considered for every task and each condition was entered as a regressor in the design matrix (AA: Sex Avoid, Sex Approach, Dance Avoid, Dance Approach; NAP: Sex Priming, Sex No Priming, Non-Sex Priming, Non-Sex No Priming; Go/No-go: Go, No-go). For each sexual task we contrasted the sexual inhibitory condition (AA: Sex Avoid; NAP: Sex Priming) against their respective control inhibitory condition (AA: Dance Avoid; NAP: Non-Sex Priming). Motion correction parameters were included as confound variables in the GLM. In separate models, we performed the same analyses including the SES and SIS scores as covariates. The resulting maps were corrected for multiple comparisons by means of cluster threshold level estimation (1000 Monte Carlo simulation iterations; Forman et al., 1995). Although, we were particularly interested in the IFG, we report whole brain analyses results since sexual inhibition neural mechanisms have been barely explored. However, for the analysis of covariance (ANCOVA) analyses, we limited the computation to the right prefrontal cortex with the aid of a mask circumscribed to this region.

### Results

#### Approach-Avoidance Task

The motivational sexual inhibition condition (Sex Avoid > Dance Avoid) led to decreased activation in the middle frontal gyrus, middle temporal gyrus, inferior parietal lobule and cuneus (Table 1; CLTC *p* < 0.005).

**Table 1:** Regions active during the avoidance of sexual stimuli.

	Motivational sexual inhibition—Approach-Avoidance task					
	BA	*x*	*y*	*z*	Size (mm^3^)	*t*
Middle temporal gyrus	21	63	−31	−2	1443	−4.55
Inferior parietal lobule	40	45	−58	43	989	−4.41
Middle frontal gyrus	10	33	62	1	664	−5.05
Cuneus	30	9	−67	7	579	−4.13

*CLTC p < 0.005*.

##### Negative Affective Priming Task

**Figure 4:**
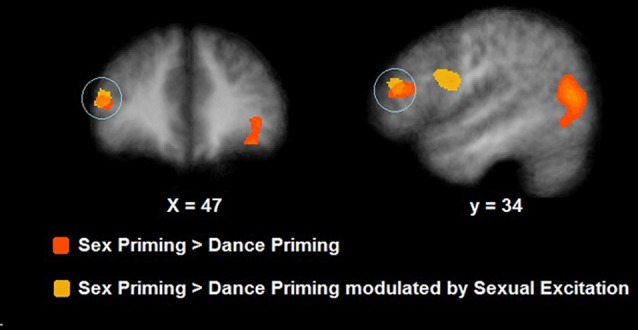
Neural activation during cognitive sexual inhibition (NAP task; in orange) and its correlates with Sexual Excitation (in yellow). During sexual priming trials, participants showed an increased activation in the right inferior frontal gyrus (IFG) which also correlated with Sexual Excitation (maps showed at a *p* = 0.01, CLTC).

During cognitive sexual inhibition (Sex Priming > Non-Sex Priming), increased activation was observed in the right inferior and middle frontal gyri, the posterior cingulate, the inferior and middle temporal gyri and the fusiform gyrus (with some areas showing also task-related activity decrease, for a complete list see Table 2; CLTC *p* < 0.005; Figure 4). To exclude the possibility that the IFG would be linked to a general sexual cognition component, we also executed the Sex No Priming > Non-Sex Priming analysis. The IFG was not activated under this condition (CLTC *p* = 0.01).

**Table 2:** Regions active during the sexual priming condition.

	Cognitive sexual inhibition—Negative Affective Priming Task					
	BA	*x*	*y*	*z*	Size (mm^3^)	*t*
Middle temporal gyrus	37	42	−64	10	6472	6.21
Inferior frontal gyrus	46	45	29	13	870	4.67
Inferior frontal gyrus	47	30	29	−12	517	5.27
Posterior cingulate	23	0	−52	22	1857	5.05
Medial frontal gyrus	10	0	56	−6	1454	4.21
Cingulate gyrus	24	−9	−4	49	305	−4.08
Precentral gyrus	6	−21	−16	64	443	−3.97
Middle frontal gyrus	11	−36	35	−9	861	4.04
Inferior temporal gyrus	19	−45	−73	1	7769	5.37
Fusiform gyrus	37	−45	−37	−11	559	4.41

*CLTC p < 0.005*.

##### Go/No-Go Task

During the inhibitory trials (No-go > Go) an increased activation was observed in the right superior frontal gyrus and bilaterally in the insula, extending to the IFG on the right side (CLTC *p* < 0.001; Table 3).

**Table 3:** Regions active during the No-go trials.

	General inhibition—Go/No-go task						
	BA	*x*	*y*	*z*	Size (mm^3^)	*t*	*P*
Claustrum	13/44	27	14	−2	5445	7.5	0.0000001
Superior frontal gyrus	9	30	50	31	1994	5.8	0.000001
Insula	13	−33	20	13	2577	7.29	0.0000001

*CLTC p < 0.001*.

#### Modulatory Role of Sexual Excitation/Inhibition Scales Scores

The SES scores of participants significantly correlated with the neural response during cognitive sexual inhibition (NAP: Sex Priming > Non-Sex Priming) within the IFG and in the middle frontal gyrus extending to the IFG (Table 4, CLTC *p* < 0.005). The latter cluster overlapped with the cluster engaged during the cognitive sexual inhibitory condition (Figure 4). The *Sexual Inhibition Scales* (SIS1 and SIS2) scores did not modulate the IFG neural response during the same condition (CLTC *p* = 0.01). The SES, SIS1 and SIS2 scores did not hold significant correlation with the IFG neural response during the sexual no priming trials of the NAP task (Sex No Priming > Non-Sex No Priming; CLTC *p* = 0.01), nor during the sexual avoiding trials of the AA task (Sex Avoid > Dance Avoid; CLTC *p* = 0.01). Unexpectedily, SIS1 scores—but not SES or SIS2—modulated the IFG (pars orbitalis) neural response during the non-sexual inhibitory trials (No-go > Go; peak values: 39, 26, 1; Size (mm^3^): 1823; CLTC *p* = 0.001). This cluster extended to the insula, where it showed an overlap with the non-modulated neural activation during the No-go trials (No-go > Go; Table 3).

**Table 4:** Sex Priming > Dance Priming correlates with Sexual Excitation scores.

	Cognitive Sexual Inhibition-Negative Affective Priming Task Neural correlates with Sexual Excitation Scale scores					
	BA	*x*	*y*	*z*	Size (mm^3^)	*r*
Inferior frontal gyrus	44	48	2	19	1060	0.74
Middle frontal gyrus	46	48	35	19	460	0.63

*CLTC p < 0.005*.

### Discussion

This study aimed at directly investigating the involvement of the IFG during sexual inhibition and the potential modulating role of individual differences in sexual excitation and/or sexual inhibition traits. To this end, we used functional neuroimaging during the execution of cognitive and motivational sexual inhibition using the established NAP and AA paradigms.

The IFG was recruited during the execution of the cognitive sexual inhibition (NAP) but not during the execution of the motivational sexual inhibition (AA) task. As expected, the IFG was also recruited during the non-sexual inhibitory Go/NoGo task. However, whereas the portion of the IFG recruited by cognitive sexual inhibition was located dorsally in the pars triangularis, the region engaged during the non-sexual inhibition was located ventrally in the opercular subdivision, in concordance with previous evidence (Dambacher et al., 2014). The disparity within the IFG regarding different types of inhibition and associated processes has been previously observed and a functional subdivision has been proposed (Cohen et al., 2013). In contrast to the non-sexual inhibition task (Go/No-go), the cognitive sexual task requires controlling bottom-up salient affective information during a cognitive process which implies more executive control and the inhibitory process occurs in an implicit rather than an explicit manner.

Unexpectedly, the IFG was not engaged during the motivational sexual inhibition task, which requires motor response control. It has been argued that the involvement of the IFG in inhibition paradigms is associated with the detection of infrequent stimuli (Erika-Florence et al., 2014). In the motivational sexual inhibition task, the inhibitory condition was presented in half of the trials which may have caused a tonic rather than an acute inhibitory demand.

We also aimed to investigate whether sexual excitation and/or sexual inhibition as traits modulated the IFG neural response during sexual inhibition processes. We observed that the degree to which an individual is, in general, more easily sexually aroused (according to SES scores) indeed significantly modulated the neural activity of the IFG during cognitive sexual inhibition but not during motivational sexual inhibition. This finding supports that individuals who are more prone to be sexually aroused have more inhibitory demands during cognitive sexual inhibition, resulting in an increased IFG activity during sexual inhibition which is modulated by sexual arousal proneness.

Sexual inhibition traits did not predict the IFG neural response during sexual inhibitory processes. Whereas the sexual inhibition traits assess the proneness to inhibit the sexual response and sexual arousal due to the threat of sexual performance, our paradigms targeted sexual inhibition at a cognitive and at motor-motivational levels. These processes may not be related at all or they may relate in other components of socioaffective cognition networks.

Although, we did not have specific predictions regarding the modulation of sexual individuals traits over the IFG neural response during general response inhibition, we observed that individuals higher in the first factor of sexual inhibition (SIS1— inhibition of the sexual response due to the threat/fear of sexual performance failure) recruited to a higher extent the IFG pars orbitalis during the No-go trials. This factor of sexual inhibition has been related to general anxiety and also proposed to be related to inhibitory tone (Bancroft et al., 2009). Both factors, anxiety and inhibitory tone could make the individual less prone to peripherically react, which would facilitate motor response inhibition but prevent the sexual response when it is desired.

In conclusion, the dorsal portion of the IFG pars triangularis (pt) showed to be specifically activated during the execution of cognitive sexual inhibition and its activity correlated with sexual excitation scores. Although these results seem to support the idea of IFGpt being an inhibitory node during cognitive sexual inhibition, the findings do not provide conclusive evidence for its causal involvement. From our correlational data we still cannot exclude alternative explanations, such as that the IFGpt processes the saliency of incoming information, increasing activity in individuals who are more easily sexually aroused when presented with sexual primed stimuli. In order to investigate whether the engagement of the IFGpt was indeed of inhibitory nature, we performed a subsequent brain stimulation experiment.

## Experiment 2: Transcranial Magnetic Brain Stimulation

In our previous neuroimaging study, we could demonstrate that the right IFGpt is engaged during cognitive but not motivational sexual inhibition. Moreover, the activation during cognitive sexual inhibition correlated with sexual excitation as a trait.

Although this region has been typically associated with inhibitory and self-regulation processes (Cohen and Lieberman, 2010; Tabibnia et al., 2011; Aron et al., 2014), it has also been argued that its involvement during inhibitory tasks maybe derived from non-inhibitory processes such as salient stimuli detection (Erika-Florence et al., 2014; Hampshire and Sharp, 2015).

To assess whether the BOLD activations that we observed were indeed associated with inhibitory processing, we employed a brain stimulation design. We tested whether the experimental deactivation of the right IFGpt using TMS would result in an experimentally-induced change in sexual inhibition capacity. Considering the specificity of its activation to cognitive sexual inhibition, we expected that brain stimulation-induced disruption would influence exclusively this inhibitory process while leaving unaffected other types of sexual inhibition or non-sexual inhibition processes. Finally, our design enabled us to experimentally assess whether brain stimulation-induced effects depend on the sexual excitation propensity of participants, considering that the level of neural activity within this region has shown to be correlated with sexual excitation as a trait during cognitive sexual inhibition (see Experiment 1).

To further increase the specificity of potential findings, we also targeted the precuneus, as this region was active during the sexual trials of both sexual inhibition tasks and did not show activation specificity for the inhibitory conditions. Thus, this region seems to play a role in sexual cognition that is not specific to the inhibitory component. Therefore, disrupting this area could modulate sexual inhibition by generally dysregulating sexual cognition in a non-specific way. To pursue our goals, we used a repeated-measures design in which participants performed the same tasks as described in Experiment 1 to target cognitive sexual and motivational sexual inhibition (NAP and AA) as well as a motor-response general inhibition task (Go/No-go) to additionally control for possible effects on a non-sexual inhibitory process.

### Method

#### Participants

Twenty-five healthy and self-reported heterosexual males were recruited for this study. One participant did not attend the third session and two participants stopped during the first session as they experienced the stimulation as uncomfortable. All participants were informed about the risks and effects of the TMS and signed an informed consent. Neither of the participants had a history of neurological disorders and all of them were right-handed (Final sample: *n* = 22; mean age = 21.8, SD = 6.25). The study was approved by the same ethical committee as in Experiment 1.

#### Design

Participants underwent three TMS sessions and an initial MRI session in case no prior anatomical MR data set was available (*n* = 15). During the TMS sessions, participants received continuous Theta burst stimulation (cTBS; Huang et al., 2005) on one of two target sites (IFGpt and precuneus) and sham stimulation; the session order was counterbalanced. Sham stimulation was delivered to the supplementary motor area, being an intermediate location between the two target sites. All sessions were scheduled at least 1 week apart from each other to diminish a learning or habituation effect. In every session, before the stimulation, participants performed few practice trials of the computerised tasks. In the first session, the individual motor threshold was determined by delivering single pulses to the right motor cortex with an increasing intensity until we observed a motor response in the left abductor pollicis brevi muscle. In each session, participants performed three computerised tasks following the cTBS stimulation (see below for details). The non-sexual task (Go/No-go) was always presented between the two sexual tasks to avoid habituation from sexual stimuli. The order of the two sexual tasks was counterbalanced across participants but the order remained constant within participants across sessions. At the end of the third session, participants were asked to fill in the same computerised questionnaires as in Experiment 1. They were identified with a number to ensure the anonymity of their responses.

#### Paradigms

As the cTBS effects start to dissipate after 45 min (Huang et al., 2005), we adapted the timing of the computerised tasks described in the “Method” section of Experiment 1 as described below. Beyond this adaptation, the tasks remained with the same characteristics.

##### Approach-Avoidance Task

The total exposition of the sexual and neutral pictures lasted for 1700 ms, and the resizing time occurred immediately after a joystick response was executed. The interstimulus fixation cross lasted for 1300, 1500, or 1700 ms. We calculated a Sex Approach-Avoid index, by subtracting the reaction times in Sex Approach blocks from reaction times in Sex Avoid blocks. A major Sex Approach-Avoid index indicated a stronger control over sexual motivation, by taking less time to avoid sexual stimuli and/or taking longer to approach them.

##### Negative Affective Priming Task

Every prime and probe trial, and every inter-trial fixation cross lasted for 1500 ms. In order to get priming effect indices for the sexual and non-sexual conditions, the reaction times in the probe trial of the No Priming condition were subtracted from the reaction times in the probe trial of the Priming condition for Sex and Non-Sex conditions. We further calculated a main sexual priming score by subtracting the non-sexual priming index from the sexual priming index. A higher index indicated a stronger sexual priming effect and thus, a stronger sexual inhibition.

##### Go/No-Go Task

Every trial consisted of the presentation of the stimulus for 200 ms, followed by an inter-trial interval of 650, 850, or 1050 ms. Responses after 650 ms with respect to stimulus onset were not registered. False alarms (responding to a No-go trial), and misses (not responding to a Go trial) were recorded.

#### Questionnaires

Participants filled in the same questionnaires as in Experiment 1.

#### Localisation of TMS Target Regions

As new participants (*n* = 19) were recruited for the present TMS study and since TMS effects are highly dependent on spatial precision, we used cortex based-alignment (CBA) for individualised TMS coil positioning. Unlike Talairach coordinates, the CBA method takes the macroanatomical differences of participants into account by aligning the individual anatomical data with pre-existing functional data in a surface space. Therefore, this method preserves a functional-anatomical correspondence, resulting in a higher spatial stimulation specificity over the region of interest and consequently stronger TMS effects (Duecker et al., 2014).

To this end, every anatomical data was segmented and a cortex reconstruction was created (80,000 vertices) from which a curvature map was extracted and transformed into a spherical space. The individual spherical space of each participant in this study was aligned to the group average spherical space from the fMRI sample proceeding from most prominent to most fine anatomical landmarks. After the alignment, the individual space was back-transformed to the individual brain anatomy and the target regions in the average surface map from fMRI were copied to the individual surface maps.

Anatomical images from the participants were collected with the same methodology as in Experiment 1.

As a target area, we selected the IFG cluster that was active during the cognitive sexual inhibition paradigm (NAP) in Experiment 1 and whose activity was shown to be modulated by the sexual excitation scores. The selection of the area was based on the surface map GLM for the Sex Priming > Non-Sex Priming contrast (*x*, *y*, *z* = 40, 28, 20). We also executed a conjunction analysis of the sexual conditions of both sexual inhibition tasks (Sex Approach ^∧^ Sex Avoid ^∧^ Sex Priming ^∧^ Sex No Priming); from the resulting map we extracted a patch of interest in the precuneus (*x*, *y*, *z* = 25, −70, 40, CLTC *p* = 0.001).

#### TMS Parameters

The TMS protocol was applied using a MagPro X100 stimulator (MagVenture A/S, Farum, Denmark) and a figure-of-eight coil (MC-B70). The co-registration of the participant’s head with their MRI anatomical data was done with the Brain Voyager TMS Neuronavigator (Brain Innovation, BV, Maastricht, Netherlands), which allowed the localisation and targeting of the regions of interest. The coil was manually held tangentially to the scalp and oriented at 45°, 90° and 45° to the central sulcus for the IFG, pre-supplementary motor area (sham), and precuneus respectively. cTBS was applied at 100% of the individual resting motor threshold (Average Maximal Stimulator Output = 32.87, SD = 3.98; Realised di/dt = 49 A/us). For the sham stimulation a placebo coil was used (MC-P-B70).

### Results

A repeated measures analysis of variance (ANOVA) revealed no main stimulation effect on sexual inhibition as there were no significant differences on the AA (*F*_(2,42)_ = 1.53, *p* = 0.22) or the NAP (*F*_(2,42)_ = 2.47, *p* = 0.09) main indexes across the three sessions. Similarly, a Friedman’s two-way ANOVA by ranks showed no differences in the number of Misses and False Alarms in the Go/No-go task (False Alarms: *p* = 0.21; Misses: *p* = 0.57). Table 5 shows the average main index for the AA and NAP tasks and the median of the frequency of False Alarms in the Go/No-go task for every session.

**Table 5:** Behavioural outcomes of the three inhibitory tasks across the three transcranial magnetic stimulation (TMS) sessions.

	Approach-Avoidance Task				
Reaction times	Sex Approach Mean (SD)	Sex Avoid Mean (SD)	Dance Approach Mean (SD)	Dance Avoid Mean (SD)	Main index Mean (SD)
Sham	784 (138)	805 (168)	819 (128)	827 (145)	−13 (82)
IFG	791 (113)	822 (131)	845 (137)	823 (115)	−52 (84)
Precuneus	808 (134)	837 (136)	862 (130)	842 (135)	−48 (119)
	**Negative Affective Priming Task**				
**Reaction times**	**Sex Priming Mean (SD)**	**Sex No Priming Mean (SD)**	**Neut Priming Mean (SD)**	**Neut No Priming Mean (SD)**	**Main index Mean (SD)**
Sham	572 (61)	579 (048)	559 (54)	575 (55)	8.6 (35)
IFG	586 (57)	579 (043)	566 (53)	581 (46)	22 (24)
Precuneus	575 (53)	582 (047)	565 (54)	573 (52)	2.3 (28)
	**Go/No-go**				
**Frequency**	**False alarms** **Median (min, max)**			**Misses** **Median (min, max)**	
Sham	0 10 (1, 36)			1.5 (0, 24)	
IFG	10.5 (0, 22)			2 (0, 8)	
Precuneus	10 (0, 28)			1 (0, 16)	

*IFG, Inferior frontal gyrus*.

**Figure 5:**
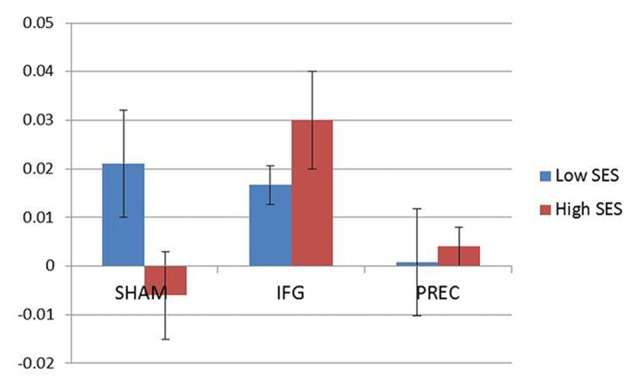
Mean indices of the cognitive sexual inhibition task (NAP) across the three sessions. For illustration purposes, the sample was median-split according to the Sexual Excitation Scale (SES) scores (Blue: low; Red: high). Participants high in sexual excitation proneness showed an increased cognitive sexual inhibition after the continuous Theta burst stimulation (cTBS) in the IFG compared to the control condition (sham) and to the cTBS in the precuneus (PREC; *F* = 4.79, *p* = 0.03).

**Figure 6:**
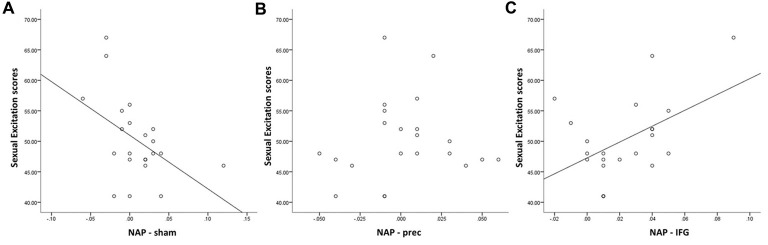
Relationship between Sexual Excitation scores and cognitive sexual inhibition (NAP index) through the three sessions. Sexual excitation negatively predicted cognitive sexual inhibition during the control condition (**A**; *R*^2^ = 0.22, *t* = −2.38, *p* = 0.03), which was reversed after the cTBS in the IFG (**C**; *R*^2^ = 0.24, *t* = 2.49 *p* = 0.04). No significant relationship was found after the cTBS in the precuneus (**B**; *R*^2^ = 0.008, *p* = 0.67).

When accounting for sexual excitation scores, however, a significant main effect of stimulation on the NAP task (*F*_(2,40)_ = 6.24, *p* = 0.004) was observed with participants showing a higher sexual priming effect after TMS over IFGpt as compared to the precuneus and the sham stimulation sessions (*p* = 0.02 and *p* = 0.05; Figure 5). No such main effects were found for the AA task (*F*_(2,40)_ = 0.72, *p* = 0.49). After adjusting for multiple comparisons, the difference between the two experimental conditions (IFGpt and precuneus stimulation) remained significant (*p* = 0.05) while the difference between the sham and the IFGpt stimulation conditions did not (*p* = 0.16). Stimulation had different effects on participants depending on their sexual excitation scores (Interaction effect: *F*_(2,40)_ = 5.73, *p* = 0.01). Regression analyses showed that higher sexual excitation scores predicted a smaller sexual priming effect during the sham session (*R*^2^ = 0.22, *t* = −2.38, *p* = 0.03; Figure 6A), but the inverse pattern was observed during the IFGpt stimulation session (*R*^2^ = 0.24, *t* = 2.49 *p* = 0.04; Figure 6C). No association was found between these two variables in the precuneus stimulation session (*R*^2^ = 0.008, *p* = 0.67; Figure 6B).

### Discussion

In this non-invasive brain stimulation study, we aimed to test whether the involvement of the right IFGpt during cognitive sexual inhibition observed in Experiment 1, was actually encoding for an ongoing inhibitory process. According to the results of Experiment 1, we expected: (a) IFGpt disruption to induce changes specifically in cognitive sexual inhibition -not in motivational sexual inhibition or general motor-response inhibition; and (b) sexual excitation as a trait to modulate these behavioural effects induced by the IFGpt disruption.

We observed that disrupting the IFGpt did modulate sexual inhibition in a specific way. Whereas brain stimulation affected cognitive sexual inhibition, it did not produce significant changes in motivational sexual inhibition or general motor-response inhibition. Importantly, this effect was significant only when taking into account sexual excitation proneness.

The present findings are relevant in two crucial ways. First, the effect was specific to cognitive sexual inhibition and did not affect other forms of inhibition. In our previous study, the different sexual inhibitory mechanisms were not related with each other behaviourally, and they distinctively predicted the frequency of different aspects of sexuality (sexual thoughts and pornography watching; Rodriguez et al., in press). Importantly, we here showed that only cognitive sexual inhibition recruited the IFGpt (Experiment 1). Second, the stimulation effect was only significant when accounting for sexual excitation which is in line with our fMRI study, in which participants with higher sexual excitation scores showed a stronger response in the IFGpt during cognitive sexual inhibition.

## General Discussion

In Experiment 1, we found that the right IFGpt is explicitly activated during cognitive sexual inhibition but not during motivational sexual inhibition or general motor-response inhibition. Supporting our hypothesis, we found that sexual excitation modulated the IFGpt activation, yet specifically during cognitive sexual inhibition. This seemed to suggest that individuals who are more easily sexually aroused have higher inhibitory resource demands which translate into more activity within IFGpt during sexual inhibition. In Experiment 2, we used TMS to disrupt the neural activity within the IFGpt and the precuneus. We observed an increase in cognitive sexual inhibition when disrupting activity within IFGpt, only when taking into account sexual excitation scores. This finding is in contradiction with the traditional role that has been adjudicated to this region because temporally disrupting the inhibitory function of rIFG with TMS should according to this classical view result in a decreased inhibition.

Although the region that we disrupted (dorsal IFGpt) has previously been engaged in inhibitory paradigms (Amin et al., 2006; Levy and Wagner, 2011), the role of the right IFG as an inhibitory module was challenged in a previous study where the manipulation of a classic inhibition paradigm (Stop Signal Reaction Time) showed that this region was not exclusively sensitive to inhibition itself but supported different processes such as the detection of salient cues and infrequent stimuli (Erika-Florence et al., 2014). This region has also shown to be relevant in visual detection of changes in the environment (Verbruggen et al., 2010) and seems to be part of the ventral attentional network which is involved in reorienting processing driven by bottom-up salient stimuli (Corbetta et al., 2008).

Remarkably, both studies (Experiment 1 and 2) were consistent in showing the modulatory effects of sexual excitation over the IFGpt during sexual cognitive inhibition. If the IFGpt is sensitive to detecting salient information, we would expect that the underlying mechanism is more prominent in individuals who are more sensitive to sexual cues during a cognitive sexual inhibition paradigm. In line with this, disrupting this process would cause a lower sensitivity to affective salient information resulting in a better affective cognitive inhibition. This reasoning is in concordance with the current results. The TMS effects were dependent on sexual excitation with individuals scoring higher in this trait showing larger benefits in cognitive sexual inhibition as a consequence of the experimentally-induced IFGpt disruption. This is, whereas individuals that scored high in sexual excitation showed less sexually cognitive inhibition during the baseline condition, this relationship was reversed when IFGpt activity was disrupted with cTBS. It can be expected that individuals who are more easily sexually aroused have a lower threshold to detect sexual cues. This salience detection mechanism interferes with their inhibitory demands. However, when this mechanism is disrupted, they can more easily ignore sexual cues facilitating their inhibitory processing.

In conclusion, this pair of studies shows that the right IFGpt is a relevant node during sexual inhibition (and in particular to cognitive sexual inhibition). However, the current evidence did not support that this region sustains the inhibitory processing, as the temporal disruption of the IFGpt produced an increase in cognitive sexual inhibition. The present evidence supports the role of the right IFGpt as a reorienting system driven by salient stimuli detection. Importantly, our study highlights the role of individual differences in psychological processes and their underlying neural mechanisms. The IFGpt activity during cognitive sexual inhibition was modulated by sexual excitation and, in line with this, the effect of TMS on the participants’ performance was also dependent on the levels of sexual excitation. These findings showed that sexual excitation proneness influence basic sexual inhibition processing and the underlying neural mechanisms. This was specific to cognitive sexual inhibition and presumably due to a higher sensitivity to sexual cues. Finally, the modulation of sexual excitation trait over the rIFG during sexual inhibition provides empirical support to the dual control model of male sexual response which states that the interplay of excitatory and inhibitory mechanisms determine the occurrence of sexual responses and associated behaviours (Bancroft and Janssen, 2000).

Deepening our understanding of the neural mechanisms underlying sexual inhibition does not only advance our knowledge on the symptomatology of sexual disorders but also provides insights for treatment improvements. In particular, the use of TMS represents an alternative to the use of medicaments to treat sexual disorders which often have undesired side effects (e.g., nausea, mood changes, bone reduction) or are contra-indicated because of the use of other pharmaceuticals. Although more research is needed, the rIFG activity could be a potential biomarker for detecting the suitability of TMS treatment. Alternatively, the modulation of cognitive sexual processes can be addressed by targeting different sub-processes such as reward processing (Prause et al., 2016). Future studies may examine the clinical suitability of the present protocol or examine different sexual inhibitory processes through different networks.

## Author Contributions

GR, AS, MD and TS made substantial contributions in the design of the study, the data interpretation and the final manuscript. GR performed the experiments and data analyses.

## Conflict of Interest Statement

The authors declare that the research was conducted in the absence of any commercial or financial relationships that could be construed as a potential conflict of interest.
